# To Sir Francis Milman, President of the Royal College of Physicians, &c. &c. &c.

**Published:** 1813-02

**Authors:** 


*To the Editors of the Medical and Physical Journal.*

GENTLEMEN,

THE underwritten is a correct copy of a letter lately presented to Sir Francis Milman. As it contains some facts intimately connected with the prosperity of the profession in general; and, as I readily believe you are actuated by the impartiality you profess, I can have no doubt but you will give it a place in your next publication.

I am, Gentlemen,
Your's, &c.
*Jan.* 14, 1813. CENSORINUS.

*To* SIR FRANCIS MILMAN, *President of the Royal College of Physicians, &c. &c. &c.*

SIR,

Having understood that an application has lately been made, by the committee acting in behalf of the general body of apothecaries, to the Royal College of Physicians, for their concurrence and support to a bill shortly to be brought into parliament for the *ostensible purpose* of regulating and improving their profession; and, as I conceive that these innovations, as far as I have come to the knowledge of them, may most materially affect the interests of that department of the healing art to which you belong, I take the liberty of offering a few observations on this subject, though I sincerely hope and trust that they may be rendered unnecessary, by the anticipations of your own superior judgment, or the suggestions of other persons more amply qualified.

It is impossible, Sir Francis, that it can have escaped your penetration, that, for a series of years, a spirit of malevolence and professional jealousy has subsisted between the physicians and apothecaries, originating, on the one hand from a persuasion that a great proportion of the prescriptions of the former are directed to the shop of the chemist; and on the other, that the province of attending on, *and prescribing for*, the sick, has been usurped and pertinaciously maintained by the apothecaries, extending in many instances to the utter exclusion of the regular physician, and consequently inducing a total incapacity on his part of providing for a family, and supporting that appearance in society which has been invariably attached to his station. Both these allegations are well founded. I have made numerous inquiries among my medical brethren, and I find that for years many of them have not sent a single prescription to the shop of those who in fact are apothecaries *only in name.* They insist that these persons, styling themselves chemists and druggists, are, according to the strict and original designation and meaning of the term, the *true* and *genuine* apothecaries, and, as such, they employ them; whilst the others, relinquishing their fair and legitimate character, endeavoring to unite the emoluments of pharmacy with the independence and respectability of prescription, and, though last certainly not least, too proud to bear the semblance of acting in a subordinate capacity, forsake those duties which they owe to the public, and ought exclusively to belong to them, to seek after others to which, by every rule of reason and justice, they are not entitled, and for which (if I might say it without offence) they are by their education totally unqualified.

Quam scit uterque libens, &c. &c.

An experience of nearly thirty years, Sir Francis, has afforded me a pretty general knowledge of the medical profession in London, and I can truly say, from my own personal conviction, that there are, in the class of apothecaries, many gentlemen of such distinguished talents and acquirements as would reflect credit on any station in life; but I am bound to add that the number of those to the general body relatively is very scanty indeed, and that at least three fourths of those who keep shops, and assume the title and discharge the functions of modern apothecaries, are as ignorant of classical learning and medical erudition, as they are of the language and doctrines of the Alcoran.

It should be recollected, that, of the four branches into which the medical profession may be divided, the surgeon-apothecaries are already in the undisputed possession of three, namely, surgery, pharmacy, and midwifery; and, so far from being satisfied, they are now making the most vigorous exertions, moving heaven and earth towards securing the same unlimited ascendency over the fourth. For this purpose, in effect, though not expressly, they have solicited the concurrence of the highly respectable body over which you preside, and also that of the College of Surgeons. Let me intreat you, Sir Francis, to reflect most seriously on this most important subject, that you will not suffer yourself to be cajoled by the specious and sophistical arguments contained in their resolutions; that you will maturely consider the the consequences of legalizing the demand of fees by a body of men whose history for many years evinces a gradual departure from their own appropriate sphere of action, and a proportionate encroachment on the practice and emolument of the regular physician; that you will call to mind how many members of your own body, owing to these causes, are struggling under difficulties and embarrassments, how many are seeking the means of a precarious subsistence in distant countries, and how many are under the necessity of quitting the profession in despair and disgust. To you, Sir Francis, whom splendid talents, or good fortune, or a happy combination of both, has so far elevated above the level of your brethren by adoption, this may appear an imaginary or exaggerated picture, but I, as well as many others, know it to be true, and could name the individuals.

It was my intention, though the application referred to at the commencement of this letter had never taken place, to have addressed you and the College in behalf of this meritorious and respectable description of men. From you they have a right to expect (as far as in you lies) an amelioration of their condition, and protection from unauthorised intrusion; whether in the shape of the sleek and pampered apothecary, or the more intolerable croud of illiterate empirics that now infest this city. No one possessed a more accurate knowledge of mankind than the great satyrist who, two thousand years ago, said

> "Sola pruinosis horret facundia pennis
> Atque inopi lingua desertas invocat artes."

Sincerely do I hope that the reproach which was thus thrown upon the Roman people, may not, in these days of British refinement, meet with a literal verification.

Among the faculty in the northern part of the United Kingdom, a plan has been for some time in agitation for physicians to keep their own medicines, and furnish them to their own patients free of expense. Upon this point a warm controversy has been maintained, by men of great experience and unquestionable talent; and the matter is still undecided. A similar idea has been entertained by several practitioners in this city, and would long since have been acted upon, but from an apprehension that it might not be sanctioned by the College. My own opinion, I confess, is adverse to the adoption of such a system, nor should it, I think, ever be resorted to but on the principle of self-defence. I have the honor of being, Sir Francis,

Your most humble and obedient Servant,

*London, Jan.* 7, 1813. CENSORINUS.

